# A Customized 3D-Printed Bolus for High-Risk Breast Cancer with Skin Infiltration: A Pilot Study

**DOI:** 10.3390/curroncol31090386

**Published:** 2024-09-05

**Authors:** Silvia Takanen, Anna Ianiro, Paola Pinnarò, Erminia Infusino, Laura Marucci, Antonella Soriani, Giuseppe Sanguineti, Giuseppe Iaccarino

**Affiliations:** 1 Radiation Oncology Department, IRCCS Regina Elena National Cancer Institute, 00144 Rome, Italylaura.marucci@ifo.it (L.M.);; 2 Medical Physics Unit, IRCCS Regina Elena National Cancer Institute, 00144 Rome, Italy; erminia.infusino@ifo.it (E.I.);

**Keywords:** breast cancer, skin toxicity, dosimetric analysis, 3D-printed bolus

## Abstract

Background: In high-risk breast cancer patients with skin infiltration, the administration of a uniform dose to superficial tissues is fundamental in order to reduce local skin relapse. A personalized bolus may prevent the potential inadequate dose distribution of a standard bolus due to air gaps between the bolus and the skin. In this pilot study, we introduced into clinical practice the use of a personalized 3D-printed bolus filled with ultrasound transmission gel. Methods: Seven patients undergoing radiotherapy after mastectomy were selected. A 3D-printed bolus dosimetric assessment was performed with MOSFET dosimeters on an anthropomorphic phantom and, subsequently, on three selected cases with increasing bolus shape irregularity. Acute/late toxicity and local control were assessed. Results: Overall, for the clinical cases, the percentage median difference between the measured and calculated doses was −2.7% (−7.0–4.9%). The median follow-up was 21 months. After two years, one patient showed G2 pain, one patient manifested G1 telangiectasia, one patient showed G1 hyperpigmentation, and two patients had no relevant toxicity. Conclusions: A personalized 3D-printed bolus filled with ultrasound gel may easily reproduce the standard bolus’ consistency and provide accurate coverage of the target area with tolerable acute/late toxicity grades. This is a pilot study, and further investigations are needed.

## 1. Introduction

In high-risk breast cancer (BC) patients undergoing mastectomy, radiation therapy (RT) plays a fundamental role in reducing the risk of loco-regional relapse (LRR) and improving overall survival (OS) [1]. The use of a slab of solid water-like material, known as a bolus, positioned at the patient’s skin is required to guarantee therapeutic dose delivery to superficial tissues, including the skin. The use of a bolus for all breast cancer patients after post-mastectomy radiotherapy (PMRT) is still debated due to skin toxicity issues, as evidenced in a variety of studies, clinician surveys, and international guidelines [2]. All these studies showed differences in bolus utilization, scheduling (i.e., daily, alternating days), materials used, and RT planning characteristics. Due to the lack of randomized trials evaluating the benefits of a bolus, a recent international consensus discussed current evidence about the use of a bolus in the setting of PMRT and its effect on LRR and toxicity [3].

In our center, we prescribe the use of a bolus in patients with skin infiltration and epidermal–dermal involvement (T4b) to cover areas of the skin and subcutaneous tissues at high risk of relapse with at least 95% of the prescribed dose [2].

Achieving the optimal fit of the bolus on the chest wall, particularly in the case of immediate reconstruction with prosthesis, is difficult. In recent years, the realization of 3D personalized bolus printing for RT has led to increasing interest in its use [4,5,6]. In this pilot study, we selected BC patients with skin involvement, with or without immediate reconstruction, and introduced a new 3D-printed bolus filled with ultrasound transmission gel to reproduce the standard bolus’ consistency and to provide adequate coverage of the target area. Its use was primarily validated on an anthropomorphic phantom with MOSFET in vivo dosimetry and then applied in clinical practice.

## 2. Materials and Methods

### 2.1. Patients

Seven patients with high-risk BC and pathological skin involvement following mastectomy with or without immediate reconstruction underwent chest-wall RT from January to November 2021. The median age was 64.5 years (range: 45–82 years).

Four patients exhibited chest-wall skin relapse after a prior mastectomy. Two patients had a postoperative pathological exam that revealed skin infiltration; one patient was staged as cT4b before neoadjuvant systemic therapy and ypT4b after surgery. Three patients underwent immediate prosthetic breast reconstruction.

RT was delivered to the target site (chest wall with or without loco-regional lymph nodes) in 25 daily fractions at a total dose of 50 Gy.

Each patient was clinically evaluated at 1 week, 3 months, 6 months, 1 year and 2 years from the end of the RT. Acute/late toxicity and local control (LC) were assessed for each patient.

### 2.2. Bolus Manufacturing Procedure

The procedure adopted for introducing the use of a personalized bolus in clinical practice was composed of the following steps, starting from computed tomography (CT) acquisition up to the bolus printing step.

CT images were acquired with a Philips Big Bore scanner (Koninklijke Philips N.V., Amsterdam, The Netherlands), with a slice thickness of 1.5 mm. The patient setup involved the use of a wing board with the patient in a supine position with their arms up. Before CT scan acquisition, four radiopaque markers were placed on the patient’s surface as reference points to longitudinally and laterally delimit the region for placing the bolus (bolus setup markers). In addition, three markers were placed at the same axial plane for the patient setup: one at the sternum location and two at the patient’s sides (patient setup markers).

CT images of the patient were imported into the Eclipse (Varian Medical Systems Inc., USA) treatment planning system (TPS), version 15.6. The body, the planning target volume (PTV) comprising the breast with or without loco-regional lymph nodes, and the main organs at risk (OARs) were contoured. The PTV was delineated to include the whole breast up to the skin.

A bolus with a thickness of 7 mm was delineated on the patient CT using the Eclipse TPS bolus tool. The primary automated shape of the bolus, created inside a virtual box, was confined to a maximum distance of 2 cm from the PTV extremities.

**Figure 1:**
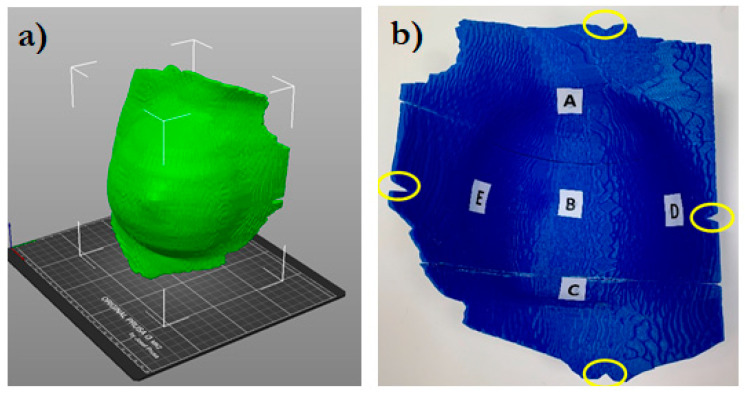
3D bolus rendering (**a**) and the inner side of the bolus (**b**). The letters A (cranial), B (central), C (caudal), D (medial), and E (external) indicate where the MOSFET dosimeters were positioned. The circles indicate the position of the cuts for the bolus alignment.

The virtual bolus structure was further modified to guide its correct positioning on the patient. Cuts were created at the position of the bolus setup markers, as shown in Figure 1.

**Figure 2:**
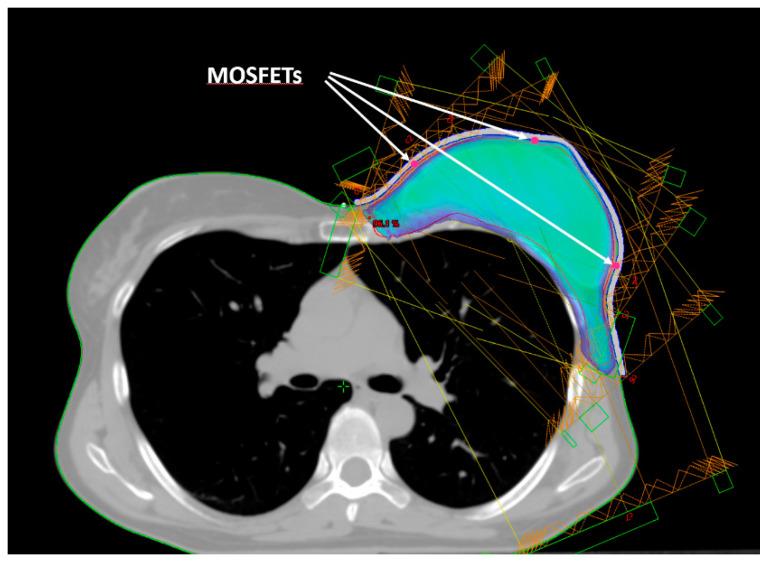
Beam arrangement for IMRT dose delivery. Isodose of 95% of the prescribed dose. The arrows indicate the positions (red dots) of the three mid-height MOSFETs (E, B, D).

Five small spherical ROIs were freehand-contoured onto the patients’ CTs at the skin–bolus interface at the positions where the dose was planned to be evaluated. The five ROIs were subtracted from the bolus ROI, generating five holes at its inner surface, which served as suitable housings for the positioned MOSFET dosimeters (Figure 2). Thus, for the following dosimetric evaluation, the position of the MOSFETs at the bolus inner surface corresponded to the ROIs contoured on the TPS. Figure 1 shows the five chosen positions: A—cranial; B—central; C—caudal; D—medial; and E—external.

The bolus structure in the DICOM format was imported into 3DSlicer [7], an open-source software that enables the conversion of the DICOM file into a standard tessellation language (STL) format. The STL file was then imported into the PrusaSlicer-2.3.3 software [8] of the Prusa i3 MK2S printer and converted into the gcode printer format.

The bolus is manufactured in polylactic acid (PLA), a fully biodegradable and nontoxic plastic with an electron density relative to water of approximately 1.1–1.2 [5]. PLA is one of the most widely used polymers in clinical applications today due to its favorable biocompatibility and to its innocuous degradation products that can safely coexist with biological systems [9]. Moreover, PLA is a readily available material with a relatively low cost with respect to a standard gel bolus.

The printing resolution (layer height) was set to 0.2 mm. The extruder and bed temperature of the 3D printer were maintained at 215 °C and 60 °C, respectively.

Boluses exceeding the maximum printing volume allowed (25 × 21 × 20 cm^3^) were printed in two or three separate pieces that were joined together using glue. Each part was printed as an empty rigid container with walls having a thickness of 1.5 mm and then filled with ultrasound transmission gel (Gel G006 ECO, Fiab, Firenze, Italy) that had a density similar to that of water (20–30 HU). Bolus walls were drilled with small holes and filled with gel using a syringe; these were then sealed with glue to prevent material leakage over the course of treatment. This technique ensured that the characteristics of attenuation and that the weight of the 3D-printed bolus were similar to those of a traditional gel bolus. A CT scan of the 3D bolus was acquired to check for the presence of accidental air bubbles.

### 2.3. Treatment Planning

The treatment technique employed for both the phantom and the patients involved four to six intensity modulated (IMRT) tangential beams with medial or external entries in the sliding window delivery mode. All the beams had a dose rate of 600 MU/min and a photon energy of 6 MV produced by a TrueBeam linear accelerator (Varian Medical Systems Inc., Palo Alto, CA, USA).

Treatment plans were optimized using the Eclipse TPS inverse-planning photon optimizer module. Dose objectives for the target and the OARs were set to meet the internal protocol tolerances. Dose calculation was performed with the Eclipse analytical anisotropic algorithm (AAA), which is a convolution–superposition-based photon-dose computation algorithm [10]. A calculation grid size of 2.5 mm was used. After dose calculation, the plans were normalized to ensure 100% of the prescribed dose to the PTV mean dose (Dmean).

The mean doses calculated in the five ROIs representing the dosimeter locations were recorded and compared with the measurements.

A cone-beam CT (CBCT) was acquired before each treatment delivery to verify the correct patient positioning and bolus fitting.

### 2.4. In Vivo Dosimetry Equipment

In vivo dosimetry was performed with MOSFET dosimeters (Best Medical International, Ottawa, ON, Canada). The choice was based on certain characteristics that make MOSFETs suitable sensors for in vivo dosimetry, such as their low energy dependence, high sensitivity, and direct reading capabilities.

Specifically, a set of dosimeters composed of three standard models (TN-502RD-H) and two microMOSFET models (TN-502RDM-H) was used. These dosimeters operated in conjunction with the Patient Dose Verification System (model TN-RD-16), using the high-sensitivity-bias supply setting. A TN-RD-38 Bluetooth transceiver was used for real-time data communication between the TN-RD-16 reader and a PC where the MOSFET software Version 2.4 was installed. The software can assign calibration factors, correction factors, and target doses to each dosimeter.

The size of the standard MOSFET and the size of the microMOSFETs are 8.0 × 2.5 × 1.3 mm^3^ and 3.5 × 1.0 × 1.3 mm^3^, respectively. The active area is 2 × 10^−5^ mm^3^ [11].

The dose delivered (cGy) is proportional to the difference in voltage shift before and after exposure (mV).

A calibration factor (cGy/mV) was determined for each MOSFET following standard procedures.

Before beam delivery, five background readings were acquired, and their average was used to correct the subsequent measurements.

### 2.5. Phantom Dosimetry

A dosimetric investigation of the 3D-printed bolus was primarily conducted on an anthropomorphic phantom, Rando^®^ (The Phantom Laboratory, Salem, NY, USA). This phantom is radiologically equivalent to an actual human body and comprises a special thermosetting synthetic rubber molded around a natural human skeleton. The phantom is equipped with two breasts composed of the same tissue-equivalent rubber, which can be secured onto its surface with plastic screws [12,13].

A treatment plan was calculated on the phantom CT as described in the treatment planning section. The plan was delivered five times with the dosimeters placed at the same positions to assess the delivery reproducibility.

### 2.6. Patient Dosimetry

MOSFET in vivo dosimetry was performed on three types of selected clinical cases, each exhibiting increasing bolus shape irregularity. The first case involved patients with chest-wall skin relapse after a prior mastectomy with an almost flat and regular bolus shape. The second case involved patients with chest-wall skin relapse after a prior mastectomy and nodal involvement, where raised post-surgery scars required the printing of an irregular-shaped bolus. The third case involved patients with prostheses after mastectomy for local relapse, for whom a bolus with a particularly convex shape was needed.

For each patient, in vivo dosimetry was performed once a week, totaling five sessions.

## 3. Results

### 3.1. Dosimetric Results

MOSFET calibration factors ranged between 0.90 and 0.93 cGy/mV, with an average of 0.91 ± 0.01 cGy/mV.

**Figure 3:**
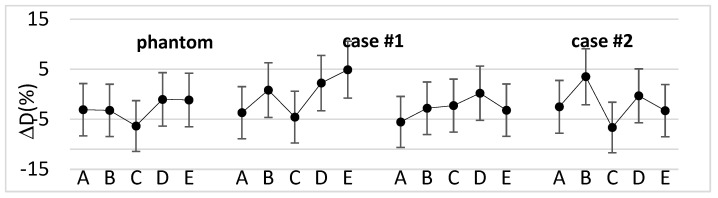
Percentage difference between the measured and calculated doses for the phantom and three types of clinical cases with increasing bolus shape irregularity at the five MOSFET positions.

Figure 3 shows the percentage difference between the measured and calculated doses for the phantom and the selected clinical cases.

Overall, for the clinical cases, the percentage difference ranged from −7.0% to +4.9%, with a median value of −2.7%. Moreover, the median absolute dose measured with the dosimeters was 47.7 Gy (44.0–50.8 Gy).

### 3.2. Clinical Results

The median follow-up (fup) was 21 months (1–26 months).

Acute and late toxicity were assessed according to the common terminology criteria for adverse effects (CTCAE) v.5.0 scale [14] and the LENT SOMA scale [15], respectively.

At 1 week after the treatment, grade 1 (G1) and grade 2 (G2) skin toxicity was observed in three and patients patients, respectively.

After three months, three patients showed G1 hyperpigmentation. One patient had no relevant toxicity. One of the patients with immediate reconstruction after mastectomy for LRR complained of G1 local pain associated with skin desquamation around the prosthesis. One patient had nodular skin relapse and died from local and distant disease progression. One patient was lost to fup.

At six months and at one year, there was no significant variation, except for the patient with a prosthesis who complained of worse G2 local pain.

Patients who developed G2 erythema experienced worse late toxicities. After one year, G2 local pain persisted uniformly in the patient with a prosthesis at the entire irradiated chest area. One patient had more evident G1 telangiectasia at the level of the upper quadrants of the chest, while one patient showed patchy G1 hyperpigmentation, and two patients had no relevant toxicity.

At two years after treatment, the patient with a prosthesis required surgical intervention to replace the prosthesis. No significant change was recorded for the remaining patients.

**Figure 4:**
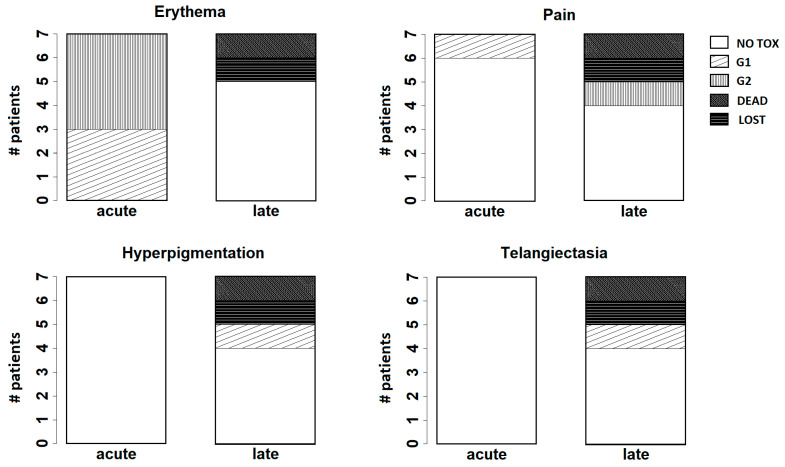
Bar plots for the main endpoints analyzed at follow-up (erythema, pain, hyperpigmentation, and telangiectasia). “Acute” refers to the results observed at the first week after treatment; “late” refers to the results observed at two years after treatment.

Figure 4 summarizes the results observed for the main endpoints analyzed at follow-up (erythema, pain, hyperpigmentation, and telangiectasia).

## 4. Discussion

In this study, the use of a 3D-printed bolus composed of a personalized-shape container in PLA filled with an ultrasound gel emulsion with physical characteristics similar to water was introduced into clinical practice in the context of a pilot study for patients undergoing mastectomy with or without immediate breast reconstruction.

The preclinical studies showed that the 3D-printed boluses seemed to overcome the disadvantages of traditionally available boluses by reducing air gaps, thus achieving doses closer to that of a uniform prescription.

Some studies [16,17,18,19,20,21] have analyzed the characteristics of different materials for 3D personalized bolus printing and are based on preliminary investigations on phantoms. Only few studies have demonstrated the efficacy of applying 3D-printed boluses to actual patients with BC. Robar et al. [5] compared the use of a 3D-printed bolus with the use of a standard SuperFlab sheet bolus for 16 patients who underwent chest-wall PMRT, demonstrating a better fitting of the 3D-printed bolus to the skin. Canters et al. [4] used a 3D shell as a mold to create a silicone bolus for the treatment of 11 non-melanoma skin cancer patients, showing that the 3D-printing process, was efficient and that the custom bolus satisfied the required dosimetric objectives.

In this study, the use of a 3D bolus filled with an ultrasound gel emulsion was primarily validated on a phantom, which assessed that the dose measured at the phantom surface with MOSFET dosimeters was in agreement with the dose calculated by the TPS. Subsequently, it was applied to patients by checking their surface dose. Figure 3 shows that, in most cases, the difference between the measured and calculated doses was within the dosimeters’ reproducibility range (±4.5%) [22] combined with the AAA dose calculation uncertainty at a 7 mm depth (±3%) [23]. The outliers can be explained considering that the dose was delivered with tangential fields and refer to points where the phantom or patient surface was steeper. However, the magnitude of the differences is in the range of the results found in previous works [5,24].

**Figure 5:**
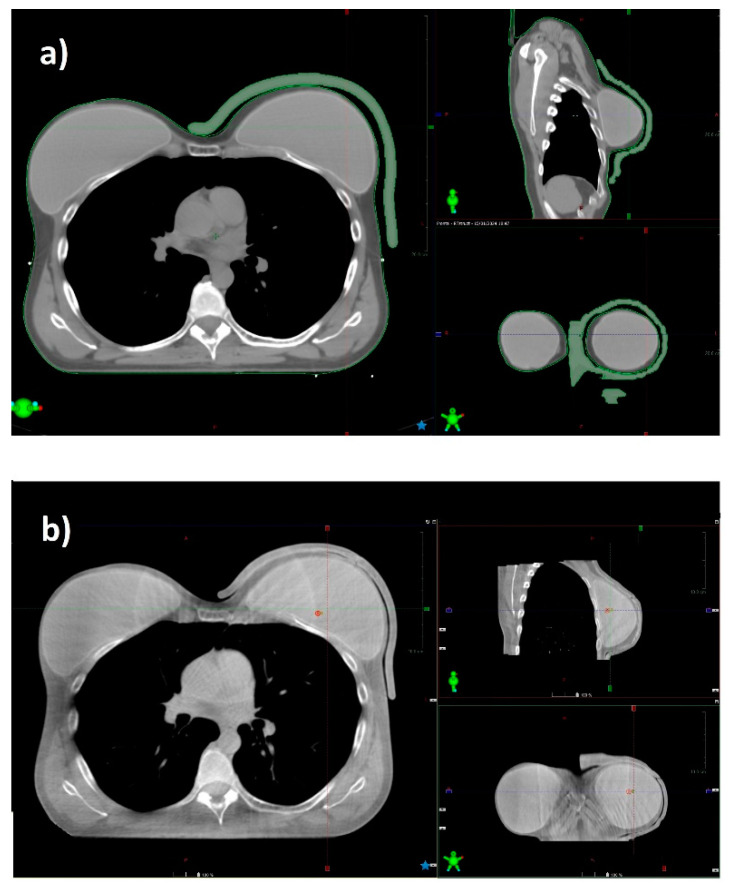
CT scan of a patient with a prosthesis and a standard gel bolus (**a**), and the CBCT acquired before radiation delivery in the same patient (**b**). The 3D bolus fits the skin without relevant air gaps.

Ensuring adequate contact with the skin using a standard bolus is challenging. Air gaps between the bolus and the skin can lead to an inadequate or inhomogeneous radiation dose delivery to the surface of the skin. At the medial and lateral borders, where the beam axis is more perpendicular to the skin, a relative dose coverage deficit near the skin is critical (Figure 5).

On the basis of the daily CBCT acquisition, we observed that the positioning of the 3D bolus is reproducible, and its shape better conforms to the skin, visibly reducing air gaps. Quantitative air gap analysis was previously described in literature [5,20]. The personalized shape of the 3D bolus makes it similar to a custom immobilization device, simplifying patient positioning, reducing setup time, and improving patient compliance. This provides significant advantages for the patient. However, the whole printing process can be time-consuming and cumbersome to manage for the operator. This represents one of the main limitations for the integration of 3D printing in a clinical workflow. In fact, the procedure to create a single 3D-printed bolus is particularly detailed and should be clearly drafted to train technical and medical physics staff. Moreover, the printing process could last up to 10–12 h depending on the printer model and bolus size. In addition, technical issues could occur, aborting the whole printing process; thus, the device should be printed in advance with a sufficient margin of time from the start of the treatment.

Regarding cost analysis, the investment of a professional device could be widely amortized over the years, considering that the cost of a PLA bolus filled with ultrasound gel is around USD 6–10.

Regarding the main outcomes, such as toxicity and LC, there is still a significant lack of data in the literature supporting the use of boluses for PMRT, with or without immediate reconstruction. A recent review [25] showed that the use of a bolus with PMRT was associated with similar LRR rates (3.5% with a bolus vs. 3.6% without) but increased acute toxicity (9.6% with a bolus vs. 1.2% without) when compared with PMRT without a bolus. The authors suggest that, in the case of skin involvement (T4b-d) or multiple high-risk features for LRR, including positive margins, extensive lympho-vascular invasion and triple-negative subtype, the use of a bolus could be recommended to achieve an adequate dose to the superficial chest-wall structures (skin and subcutaneous tissue). Nichol et al. [6] observed that, after mastectomy, the use of a bolus doubled the risk of G2 and G3 skin toxicity and, upon multivariable analysis, its use was not associated with better LC. Anderson et al. [24] analyzed the complications and cosmetic results related to the use of a custom wax bolus fitting the skin, showing that a customized bolus can significantly reduce the incidence of complications compared with traditional bolus sheets (9% vs. 24%).

Both the traditional bolus and the 3D-printed bolus not only increase the irradiation dose on the skin surface, but they also aggravate the skin reaction to varying degrees. However, interpretation of the available pooled toxicity data in the literature is limited by the variation in toxicity scales used across studies. In particular, little is known about the use of a bolus in the hypofractionation setting, immediate breast reconstruction [26], and the different types of breast reconstruction (e.g., autologous vs. implant) in terms of LRR, toxicity, and reconstruction-related complications [25].

In our pilot investigation, we reported G2 radiation dermatitis as the maximum and more frequent acute side effect. At the two-year follow-up, the maximum late toxicity was G2 pain in one patient with a prosthesis, G1 telangiectasia in one patient, and G1 hyperpigmentation in another patient. Further investigations with a higher number of patients are needed. Moreover, to evaluate the efficacy in terms of LC of the 3D-printed bolus filled with an ultrasound gel, a longer fup is required.

## 5. Conclusions

The development of customized 3D-printed boluses has been shown to reduce the air gap, improving the accuracy and uniformity of the dose and better protecting normal tissues. However, there is a lack of scientific evidence regarding the use of a 3D bolus, and there is not yet a consensus about the material choice and frequency of application. Our pilot study showed that a personalized 3D-printed bolus filled with ultrasound gel may easily reproduce the standard bolus’ consistency and provide accurate coverage of the target area, especially in post-mastectomy radiotherapy with prosthesis, with tolerable acute/late toxicity grades. Further investigations with a higher number of patients and a longer fup are needed.

## Data Availability

The data presented in this study are available in this article.
